# NLRP3 inflammasome activation in response to metals

**DOI:** 10.3389/fimmu.2023.1055788

**Published:** 2023-02-10

**Authors:** Wanyi Huang, Ziqi Zhang, Yueyang Qiu, Yuan Gao, Yongqiang Fan, Qiang Wang, Qing Zhou

**Affiliations:** ^1^ School and Hospital of Stomatology, China Medical University, Shenyang, China; ^2^ Liaoning Provincial Key Laboratory of Oral Diseases, China Medical University, Shenyang, China; ^3^ Department of Orthodontics, Shenyang Stomatological Hospital, Shenyang, China; ^4^ College of Life and Health Sciences, Northeastern University, Shenyang, China; ^5^ Shenyang National Laboratory for Materials Science, Northeastern University, Shenyang, China

**Keywords:** inflammasome, metal, inflammation, implant, osteoimmunology

## Abstract

Implant surgery is followed by a series of inflammatory reactions that directly affect its postoperative results. The inflammasome plays a vital role in the inflammatory response by inducing pyroptosis and producing interleukin-1β, which plays a critical role in inflammation and tissue damage. Therefore, it is essential to study the activation of the inflammasome in the bone healing process after implant surgery. As metals are the primary implant materials, metal-induced local inflammatory reactions have received significant attention, and there has been more and more research on the activation of the NLRP3 (NOD-like receptor protein-3) inflammasome caused by these metals. In this review, we consolidate the basic knowledge on the NLRP3 inflammasome structures, the present knowledge on the mechanisms of NLRP3 inflammasome activation, and the studies of metal-induced NLRP3 inflammasome activation.

## 1 Introduction

Implantation has been widely used in orthopedic and dental treatments to replace the non-regenerative part of the human body (1, 2). Surgical implantation is accompanied by hemorrhage, which causes various cells and proteins between the bone and surrounding soft tissue to participate in a series of biological processes, including protein deposition, coagulation, inflammation, and tissue formation (3). Leukocytes migrate into the peri-implant site, and the activation of leukocytes results in the release of inflammatory mediators, including interleukin-1 beta (IL-1β), IL-6, tumor necrosis factor alpha (TNF-α), and macrophage colony-stimulating factor (3). The initial reaction of inflammatory cells to foreign materials influences the bone healing process. Thus, it is essential to study biomaterial-induced inflammation in the osteogenesis field (4). The inflammasome is an essential part of the innate immune system that is involved in metal-induced hypersensitivity, bacterial infection-induced peri-implantitis, and other possible side effects of implantation (5, 6). The activation and assembly of the inflammasome are complicated programmed processes that involve the upstream sensors, the adaptors, and the downstream effectors (7). Therefore, studies on implantation-induced inflammasome activation have constantly increased over the years.

Broadly, biomaterials are grouped into natural precursors or synthetic materials (8, 9). Suitable implant materials must satisfy the biochemical, physiological, and antibacterial property requirements of implantations. The implant surface chemistry and topography influence the process of osteogenesis (10). There are two main methods to improve osseointegration: 1) sintering of the metallic beads or fibers over the implant surface and 2) plasma spray deposition of the metallics or ceramics onto the implant surface (3). Metal implants apply load-bearing sections, such as in long bone and dental implants. Metal implantation can constantly precipitate metal ions, which are components of cellular proteins, bone structures, and intracellular or extracellular matrices (11). In addition, the release of metal ions into the surrounding bone tissues participates in osseointegration, which has been used to describe the successful healing of an implant within a host bone (12).

Peri-implant tissue healing starts with an inflammatory response after the implant is inserted into the bone cavity (13); however, inflammation at the peri-implantation site is also the leading cause of implantation failure (14). Therefore, it is essential to study the immune response to implant biomaterials. However, the role of implant-released metal ions in inflammasome activation, a critical component of the host immune system, is still unclear. Recent studies on metal-activated inflammasomes have mainly focused on the NOD-like receptor protein-3 (NLRP3) inflammasome. In this review, we first present an overview of the series of NLRP3 inflammasome activation mechanisms and then summarize recently published research on various metal ions, metal particles, and metalloproteins that activate the NLRP3 inflammasome signals.

## 2 NLRP3 inflammasome

The inflammasome, identified by Tschopp and co-workers in 2002, is described as a high-molecular-weight complex present in the cytosol of stimulated immune cells that mediates the activation of inflammatory caspases (15). The activated caspases convert IL-1β and IL-18 from their inactive to their active forms. At the same time, full-length gasdermin D (FL-GSDMD) is cleaved to the N-terminal GSDMD (GSDMD-NT) and forms membrane pores, resulting in cytokine release and/or programmed cell death called pyroptosis (16, 17). The inflammasomes are named after the pattern recognition receptors (PRRs) that sense the pathogen-associated molecular patterns (PAMPs) and the damage-associated molecular patterns (DAMPs) in the cytosol and initiate downstream responses. In general, inflammasomes are classified into canonical and non-canonical inflammasomes.

Canonical inflammasomes comprise a sensor molecule, the adaptor ASC (apoptosis-associated speck-like protein containing a C-terminal caspase recruitment domain (CARD), and the effector caspase-1. Several sensors have been identified. There are sensors that consist of the nucleotide-binding oligomerization domain (NOD) and leucine-rich repeat (LRR)-containing protein (NLR) family members, including NLRP1, NLRP3, NLRC4, NLRP6, NLRP7, and NLRP12. These sensors, which activate inflammasomes, are classified as NLR N-terminal domains. Another class of inflammasomes assembling sensors is represented by the PYHIN protein family members, such as melanoma 2 (AIM2) and pyrin (7). The ASC adaptor contains two death-fold domains: one pyrin domain (PYD) and one caspase recruitment domain (CARD). On the one hand, PYD forms PYD–PYD interactions with the PYD domain in activated sensors. On the other hand, the CARD domain interacts with the CARD domain in pro-caspase-1. Therefore, ASC serves as a bridge between the sensors and pro-caspase-1, forming inflammasomes as a result of recognition of the PAMPs and DAMPs in canonical inflammasomes (6).

**Figure 1:**
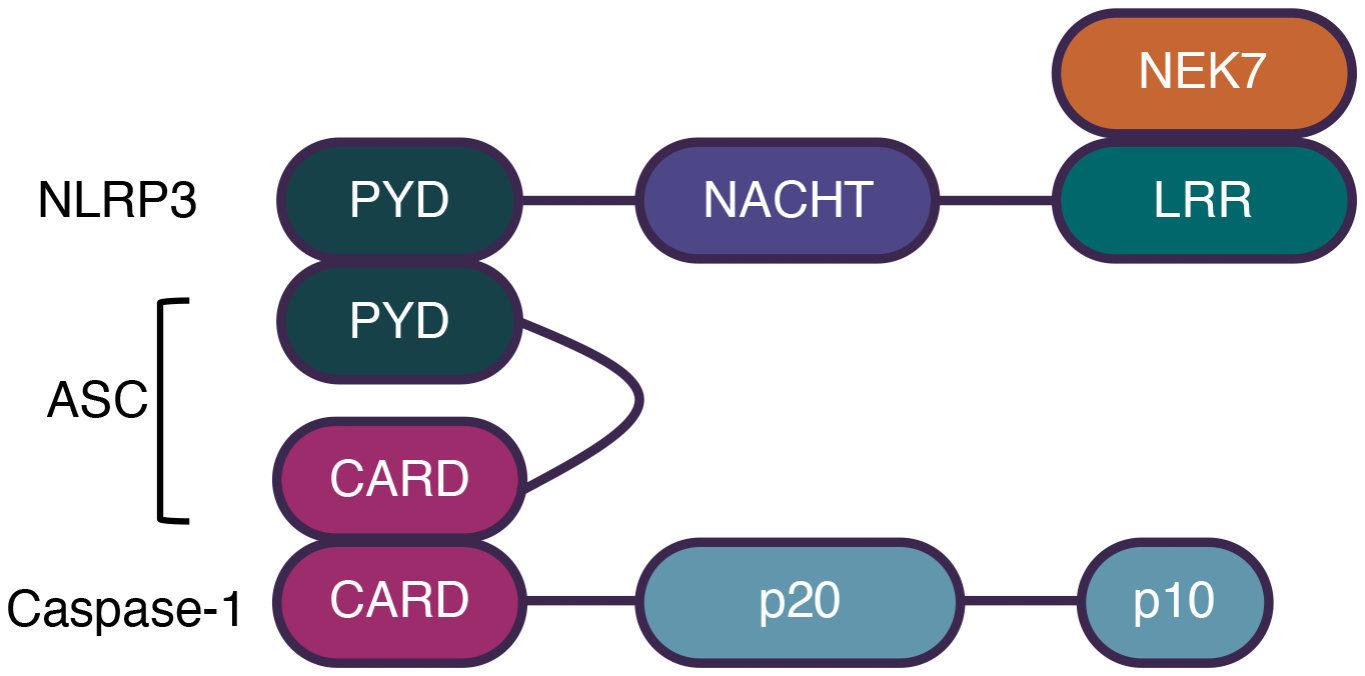
The structure of NLRP3 inflammasome. Canonical inflammasomes comprise a sensor molecule, the adaptor ASC, and the effector Caspase-1. NLRP3 comprises a N-terminal PYD, a central NOD, and a C-terminal LRR domain. NEK7 interacts with LRR domain to promote NLRP3 activation. ASC comprises a PYD and a CARD domain. Caspase-1 comprises a CARD and two subunits, p10 and p20. Sensors interact with ASC by PYD domains and ASC interacts with caspases-1 by CARD domains.

The NLRP3 inflammasome plays critical roles in various inflammatory disorders, including Alzheimer’s disease, diabetes, and other inflammation-related diseases (18–20). It consists of an N-terminal PYD, a central NOD (also called the NACHT domain), and a C-terminal LRR domain (21). The ATPase activity of NOD is essential for NLRP3 oligomerization and is targeted by MCC950, a commonly used NLRP3 inhibitor (22). In addition, it has been determined that the NIMA-related kinase 7 (NEK7) interacts with the LRR domain to promote the activation of NLRP3 (23). The structure of the NLRP3 inflammasome is shown in **Figure 1**.

**Figure 2:**
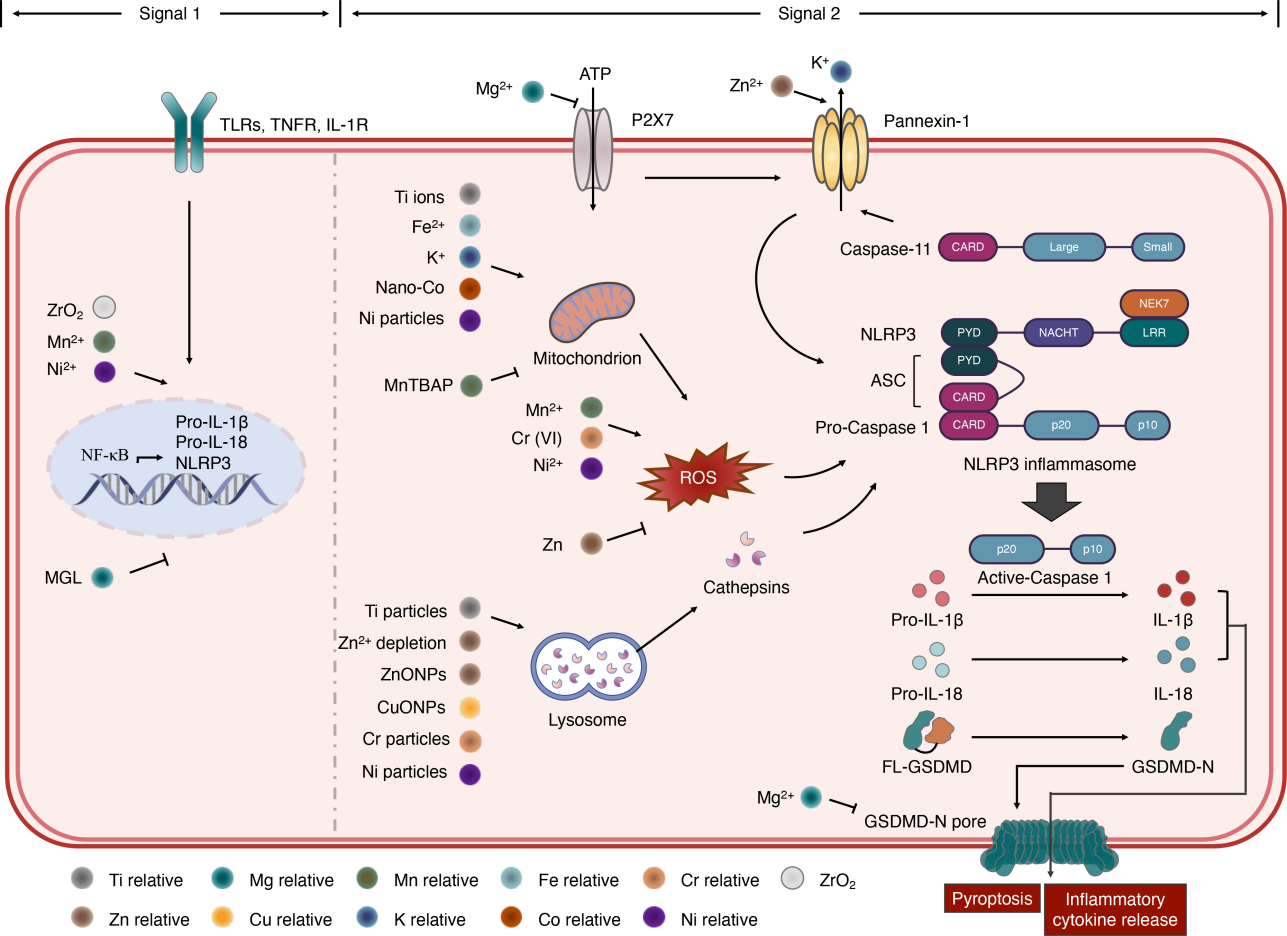
Roles of metals in NLRP3 inflammasome activation. NLRP3 inflammasome activation requires a two-step process. In signal 1, NF-κB transcriptional activation can be accelerated by Mn^2+^ binding prion fibrils, Ni^2+^ ions, ZrO_2_ and inhibited by magnesium isoglycyrrhizinate (MGL). In signal 2, ionic flux activating NLRP3 inflammasome is controlled by the P2X7 and pannexin-1 receptors. The pannexin-1 activity can be regulated by Zn^2+^. Mg^2+^ can inhibit the ATP-gated Ca^2+^ channel P2X7 and limits the oligomerization and membrane localization of GSDMD-NT, which form the GSDMD-N pore. In addition, caspase-11 cleave pannexin-1, resulting in K^+^ efflux and NLRP3 inflammasome activation. Mitochondrion dysfunction and ROS production can induce NLRP3 inflammasome activation. Ti ions, Fe^2+^, K^+^, Nano-Co and Ni particles promote this process, while MnTBAP inhibits it. Zinc can attenuate ROS production while Mn^2+^, Cr (VI) and Ni^2+^ induce the release of ROS, triggering NLRP3 inflammasome. Ti particles, Zn^2+^ depletion, ZnONPs, CuONPs, Cr pariticles and Ni particles can induce lysosome damage, which induced cathepsins release and result in NLRP3 inflammasome activation.

The NLRP3 inflammasome has been extensively studied for its broad spectrum of stimuli and complicated activation signaling. The stimulators of NLRP3 include crystalline materials, extracellular ATP, pore-forming toxins, RNA–DNA hybrids, peptide aggregates, and viral, fungal, and bacterial pathogens (6, 24). It is generally accepted that the activation of the NLRP3 inflammasome involves a two-step process. Signal 1, also known as the priming signal, is triggered by PRR signaling, such as Toll-like receptors (TLRs) or cytokine receptors, e.g., TNF and IL-1 receptors. The activation of signal 1 leads to the transcriptional activation of nuclear factor kappa B (NF-κB), which upregulates the gene expression of NLRP3, IL-1β, and IL-18. Signal 2, also known as the activation signal, is induced by various PAMPs and DAMPs. Multiple molecular and cellular signaling events have been proposed to activate the NLRP3 inflammasome, as follows: 1) ionic flux, including K^+^ efflux, Ca^2+^ mobilization, Cl^−^ efflux, and Na^+^ influx. As a DAMP, ATP activates the P2X7 receptor on the cell membrane, inducing K^+^ efflux that triggers the activation signal; 2) mitochondrial dysfunction and the production of reactive oxygen species (ROS); and 3) lysosome damage. However, none of these is recognized as a common event induced by all the NLRP3 inflammasome stimuli (21, 25). To date, studies on metal-activated inflammasomes have mainly focused on the NLRP3 inflammasome (26, 27). Details of these studies are discussed in the following sections and are summarized in **Figure 2**.

## 3 Metals involved in NLRP3 inflammasome activation

As metals are the primary implant materials, metal-induced local inflammatory reactions have received significant attention, and there has been more and more research on the activation of inflammasomes caused by metals (28, 29). One of the present trends of biomaterials is the development of strategies and solutions that modulate the immune cell biology to avoid or minimize the undesired side effects in regenerative medicine (30). In the host response to biomaterials, the inflammasome is the bridge between inflammation and tissue regeneration. As the widest studied inflammasome, the NLRP3 inflammasome has become a major topic in the development of immunomodulatory biomaterials. Modulation of NLRP3 inflammasome activation in response to biomaterials in order to minimize the tissue inflammatory response to implants can promote tissue regeneration around the implant and determine the outcomes post-implantation (31). The reaction and mechanisms of metals activating the NLRP3 inflammasome depend on the different metal elements and metal states. We summarize recent studies on the roles of metals in NLRP3 inflammasome activation in **Table 1**. Details of specific metals are discussed as follows.

**Table 1:** Present studies regarding the roles of metals in NLRP3 inflammasome activation.

Activator	Mechanism	Species	Model	Reference
Ti ions	Promote ROS production	Human	Jurkat T cells	(32)
Ti particles	Lysosome and lysosomal cathepsins	Mice, human	Primary macrophages, THP-1 cells	(33)
Ti nanoblets	Lysosome membrane permeabilization increase and cathepsin leakage	Mice	Caspase-1-deficient mice, mouse bone marrow derived macrophages (BMDMs)	(34)
Ti particle	GSK-3β/β-catenin pathway	Rats	Male Sprague–Dawley rats, rat mesenchymal stem cells (rMSCs)	(35)
Zinc ions	Inhibit the activity of pannexin-1	Mice	Primary macrophages	(36)
TPEN	Damage the integrity of lysosome	Mice	Primary macrophages, J774 cells	(37)
Zinc ions	Inhibit high glucose-induced NLRP3 inflammasome activation by attenuating ROS production	Human	Human peritoneal mesothelial cell line HMrSV5	(38)
Zinc gluconate	Suppress NLRP3 inflammasome by promoting autophagy and ubiquitination	Mice	C57BL/6J mice, BV2 cells (a microglia cell line)	(39)
Zinc gluconate	Regulate miR-374a-5p and promote microglial autophagy-induced NLRP3 inflammasome inactivation	Mice	C57BL/6J mice, BV2 cells	(40)
TPEN	Unknown	Mice	Primary astrocytes, BV2 microglial cells	(41)
ZnONPs	NLRP3 inflammasome–autophagy–exosomal pathway	Mice, human	Human keratinocyte HaCaT cells, SKH-1 hairless mice	(42)
MgCl_2_, MgSO_4_, MgGluc_2_	Inhibit the ATP-gated Ca^2+^ channel P2X7	Mice, human	HEK 293T cells, 293FT cells, THP-1 cells, primary immortalized BMDM cells (iBMDMs), LPS (intraperitoneally) mice	(43)
MGL	Unknown	Rats	Fructose-fed Sprague–Dawley rats with metabolic syndrome	(44)
MGL	Unknown	Mice	Con A-induced liver injury mouse model	(45)
Copper depletion (TTM)	Remove copper from the active site of superoxide dismutase 1	Mice, human	SOD1-, NLRP3-, and caspase-1/-11-deficient mice, human macrophages from ascite fluid, human blood-derived monocytes	(46)
CuONPs	Induce lysosomal damage along with the release of cathepsin B, release Cu^2+^, and induce cellular oxidative stress	Mice	J774A.1 macrophage	(47)
Multi-target iron chelator M30	Inhibit activation of the AC/cAMP/PKA/HIF-1*α*/NLRP3 pathway	Rat	Hepatocyte cell line BRL-3A	(48)
Ferric ammonium citrate	Cellular labile iron induces ROS production and mitochondrial dysfunction	Human	PBMCs, THP-1 (human monocyte) cells	(49)
MnCl_2_	Induce ROS release	Mice	Oropharyngeal aspiration exposed mice, HAPI cells	(50)
Mn	Mitochondrial defects	Mice	Oropharyngeal aspiration exposed mice, primary microglial cells	(51)
MnCl_2_	Mn^2+^ binding on prion fibrils	Mice	immortalized mouse brain EOC 13.31 microglial cells	(52)
MnSOD	Enhance ROS scavenging ability	Mice	Isolated heart perfusion	(53)
MnTBAP	Block albumin-induced mitochondrial dysfunction	Mice	Intraperitoneal injection exposed mice, mouse proximal tubular cells (mPTCs)	(54)
MnTE-2-PyP	Unknown	Mice	Hypoxic mouse model	(55)
Mn-TAT PTD-Ngb	Enhance ROS scavenging ability and abate mitochondrial dysfunction	Mice	BV2 cells	(56, 57)
KCl	NLRP3–NEK7 interaction	Mice	Nek7^−^, Nlrp3^−^, Asc^−^, Casp1/Casp11^−^,Casp11^−^ deficient mice, BMDMs	(58)
Indanyloxyacetic acid-94 (IAA94)	Induce mitochondrial dysfunction and mtROS production	Human	THP-1 cells	(58)
KCl	P2X7 receptor	Mice	Casp1/Casp11^−^, Casp11^−^, Nlrp3^−^, Nlrp6^−^, Nlrp12^−^, Nlrc4^−^, Pycard^−^, P2x7^−^, Panx1^−^, and AIM2^−^ deficient mice	(59–61)
Cr(VI) compounds	Induce mtROS production	Mice, human	Primary human monocytes, primary human keratinocytes, murin dentritic cells	(62)
Cr particles	Lysosome–cathepsin B	Human	Primary macrophage	(63)
Cr^3+^	Unknown	Mice	BMDMs	(28)
CoCl_2_	Unknown	Human	HaCaT cells	(64)
CoCl_2_	Negative regulation by inducing hypoxia	Mice	BV-2 cells and primary mixed glial cells	(65)
Nano-Co	Promote intracellular oxidative stress damage and mitochondrial reactive oxygen species (mtROS)	Human	Liver L02 cells	(66)
CoPP	Reduce the amount of intracellular ASC	Mice, human	Human primary macrophages, THP-1 cells, BMDMs	(67)
Ni particles	Decrease MMP and increase MPTP, inducing mitochondrial dysfunction and ROS production, induce the Warburg effect	Human	Human lung epithelial BEAS-2B cells	(68, 69)
NiCl_2_·6H_2_O	Phagolysosome–cathepsin B pathway	Mice	BMDMs, bone marrow dendritic cells (BMDCs)	(70)
Ni particles	Disrupt phagolysosome	Mice	BMDMs, oropharyngeal aspiration exposed mice	(71)
NiCl_2_	Mitochondrial dysfunction, mtROS production, mtDNA release	Mice	BMDMs	(72)
ZrO_2_	TLR4	Human	THP-1 cells	(73)
Al_2_O_3_	Unknown	Mice	Intraperitoneal injection exposed mice	(74)

TPEN, N,N,N′,N′-tetrakis(2-pyridylmethyl)ethylenediamine; ZnONPs, zinc oxide nanoparticles; MGL, magnesium isoglycyrrhizinate; TTM, tetrathiomolybdate; CuONPs, copper oxide nanoparticles; MnSOD, manganese superoxide dismutase; MnTBAP, manganese tetrakis porphyrin chloride; PTD-Ngb, protein transduction domain–neuroglobin; CoPP, cobalt protoporphyrin; ROS, reactive oxygen species; ASC, apoptosis-associated speck-like protein containing a C-terminal caspase recruitment domain; MMP, mitochondrial membrane potential; MPTP, mitochondrial permeability transition pore; mtDNA, mitochondrial DNA.

### 3.1 Titanium

Titanium (Ti) and its alloys have recently attracted significant interest (75). They are the most widely used metals for implantation due to their advantageous characteristics, including their excellent corrosion resistance and bone-bonding ability (76). The interface between metals and the surrounding tissues is also critical as insufficient bonding provides a bacterial invasion route, resulting in peri-implantitis. Titanium ions are frequently detected in the implant region, especially in peri-implantitis tissue (75). It has been reported that Ti ions can promote the production of ROS, and *N*-acetyl-l-cysteine (NAC), a ROS scavenger, decreased the Ti ion-induced NLRP3 gene expression and IL-1β release, suggesting that Ti ions activated the NLRP3 inflammasome in an ROS-dependent manner (32).

However, research has shown that Ti ions alone cannot stimulate the transcription of the inflammasome components, but they form particles that stimulate inflammasome activation and, consequently, IL-1β release (77). It is widely accepted that Ti particles can activate the NLRP3 inflammasome (78, 79). However, the mechanisms of Ti-induced inflammasome activation in inflammatory diseases are still controversial. Several studies have suggested that Ti particle-induced activation of the NLRP3 inflammasome is dependent on lysosomes and lysosomal cathepsins. St. Pierre et al. found that the macrophage uptake of Ti particles was cathepsin B-dependent and induced acute inflammation by activating the NLRP3 inflammasome, resulting in IL-1β release and neutrophil recruitment (33). In a rat peri-implant osteolysis model, Ti particles induced NLRP3 inflammasome activation depending on mitochondrial function. Sirtuin 3, an NAD^+^-dependent deacetylase of the mitochondria that regulates its function, suppressed the Ti particle-induced NLRP3 inflammasome activation *via* the GSK-3β/β-catenin pathway (35). In addition, Ti particles can also induce cell death. However, NLRP3 and gasdermin D did not participate in the cell death process, suggesting that the Ti particle-induced cell death was not pyroptosis (80). An *in vitro* study also showed that Ti particles alone were insufficient at inducing the IL-1β release in macrophages; an additional priming signal, such as bacterial lipopolysaccharide (LPS), was required to enable inflammasome activation (63). Therefore, reducing the amount of particles produced in the process of implant surgery and application is critical to suppressing the activation of the NLRP3 inflammasome.

### 3.2 Zinc

Nearly 90% of zinc (Zn) is found in muscles and bones. Zn has a stimulatory effect on bone metabolism and the ability to promote bone formation and mineralization (81). It is accepted that the immune system is regulated by Zn homeostasis, and Zn^2+^ functions as a second passenger in innate immunity (27). Zn homeostasis is maintained by the Zn^2+^ transporter proteins, including SLC30A (ZnT) and SLC39A (ZIP, Zir/Irt-like proteins) (82). The effects of Zn in NLRP3 inflammasome activation have been proven in series studies. Brough et al. explored pretreating the macrophage with *N*,*N*,*N*′,*N*′-tetrakis(2-pyridylmethyl)ethylenediamine (TPEN), the Zn chelator, and found significant inhibition of the activity of pannexin-1, thus suppressing the activation of the NLRP3 inflammasome and decreasing the production of IL-1β (36). Pretreatment of primary mouse macrophages with TPEN also damaged the integrity of the lysosome, thus suppressing the NLRP3 inflammasome activation and IL-1β secretion (37). In addition, Zn can inhibit high glucose-induced NLRP3 inflammasome activation in peritoneal mesothelial cells by attenuating ROS production (38). Zn also played a neuroprotective role by suppressing NLRP3 inflammasome activation through autophagy and ubiquitination in an experimental spinal cord injury model (39, 83). Another study also showed that Zn suppressed NLRP3 activation by inducing microglia autophagy and played a neuroprotective role in spinal cord injury (40). Moreover, Zn participated in LPS and hypoxia inducing NLRP3 inflammasome activation in the microglia (41). Zinc oxide nanoparticles (ZnONPs) can suppress the NLRP3 inflammasome using the NLRP3 inflammasome–autophagy–exosomal pathway (42).

### 3.3 Magnesium

Magnesium (Mg) is a degradable and absorbable biomaterial. It is widely used in the clinic as its structural and mechanical characteristics are similar to those of the trabecular bone, which is beneficial to obtaining early fixation (84). Mg homeostasis in cells is maintained by Mg channels and transporters, including Mrs2, SLC41A1, SLC41A2, and TRPM6 (82).

It has been reported that Mg ions can suppress both the canonical and non-canonical pyroptotic pathways in macrophages by inhibiting the ATP-gated Ca^2+^ channel P2X7 and limiting the oligomerization and membrane localization of GSDMD-NT, thus blocking the GSDMD-NT-induced pyroptosis (43). In addition, magnesium isoglycyrrhizinate (MGL), a new stereoisomer of glycyrrhizic acid, performs immune modulation through its anti-inflammatory effect and is clinically used as a hepatoprotective agent for the treatment of liver diseases. The anti-inflammatory effects of MGL may involve suppressing the activation of inflammasomes (44, 45, 85). MGL inhibited both NF-κB activation and NLRP3 inflammasome formation, thus alleviating liver inflammation in fructose-fed rats with metabolic syndrome (44). MGL was also used to treat chronic obstructive pulmonary disease by suppressing NLRP3 and cleaving caspase-1 expression (85). In addition, MGL administration can decrease the expression of NLRP3, NLRP6, and caspase-3 in mice, suggesting its downregulatory inflammasome expression effect in liver tissue (45).

### 3.4 Copper

Copper (Cu), an indispensable trace element in organisms, plays a crucial role in a lot of physiological activities, including respiration, iron metabolism, antioxidant activity, and tissue integrity (86). It is well known that the addition of Cu can endow biomaterials with antibacterial properties, osteogenesis, and angiogenesis (87). In organisms, Cu exists in two states: Cu(I)/Cu^+^ (cuprous ion) and Cu(II)/Cu^2+^ (cupric ion). Cu^2+^ is the predominant redox state in blood, whereas Cu^+^ is the form found in the reducing environment of the cell cytosol (88). Cu^2+^ can be reduced to Cu^+^ through the plasma membrane reductase of the STEAP (six-transmembrane epithelial antigen of prostate) family or DCYTB (duodenal cytochrome B) and then transported into cells by CTR1 (copper transporter 1) or DMT1 (divalent metal ion transporter 1), which are located in the membrane (89). In addition, proteins called metallochaperones also distribute Cu to specific sites in cells (90).

The dyshomeostasis of Cu has been reported to trigger inflammasome activation. Metal chelators remove metal ions from the body, reducing the metal concentration. Tetrathiomolybdate (TTM) is a highly specific, clinically approved Cu chelator (91) that can be used as an anti-inflammatory agent to prevent LPS-induced inflammatory reactions *in vivo*. Deigendesch et al. showed that TTM could prevent the activation of NLRP3 by removing Cu from the active site of superoxide dismutase 1 (SOD1) in macrophages. This regulation targets macrophages, not monocytes, in both mice and humans (46). In addition, TTM did not block the NF-κB and mitogen-activated protein kinase (MAPK) pathways or the other major antimicrobial inflammasomes such as NLRC4, NLRP1, and AIM2 (44). *In vivo*, depletion of bioavailable Cu led to a decreased caspase-1-dependent inflammation and reduced the susceptibility to LPS-induced endotoxic shock (44). Exposure to copper oxide nanoparticles (CuONPs) also resulted in NLRP3 activation by inducing lysosomal damage and the release of cathepsin B. Moreover, after lysosomal deposition, CuONPs released Cu^2+^ due to the acidic environment of lysosomes. Cu^2+^ then induced cellular oxidative stress and further mediated the activation of the NLRP3 inflammasome (47).

### 3.5 Iron

Iron (Fe) is involved in many critical biological processes, such as oxygen transport, ATP generation, and DNA biosynthesis. With regard to tissue engineering, Fe has excellent mechanical properties, making it a good candidate for implants requiring high structural strength, such as bone defect repair and vascular stents (92). Fe has redox activity, and a high Fe level can induce ROS, leading to oxidative stress and signal pathways critical to cell survival and death (93). The homeostasis of Fe metabolism in the human body is strictly regulated by a variety of proteins, including, among others, ferritin (FTH1 and FTL), a protein complex that stores Fe in cells for future use; transferrin (TF), an Fe-binding serum protein; transferrin receptor 1 (TfR1, *TFRC*), a plasma membrane protein that allows cells to ingest transferrin; divalent metal transporter 1 (DMT1, *SLC11A2*), a critical metal transporter for TfR1-mediated Fe uptake and dietary Fe absorption; and ferroportin (Fpn, *SLC40A1*), the only known cell Fe efflux pump (94).

The multi-target Fe chelator M30 has an Fe chelating/free radical scavenging effect. It inhibits lipid peroxidation, which has been proven to inhibit oxidative stress and inflammation in many diseases, such as type 2 diabetes and Alzheimer’s disease. In an *in vitro* ethanol-induced hepatocyte injury model, M30 inhibited the activation of the AC/cAMP/PKA/HIF-1/NLRP3 inflammasome pathway, ameliorated oxidative cell stress, and reduced cell damage (48). Fe^2+^-specific chelators can also rescue peripheral blood mononuclear cells from an LPS stimulation-induced Fe^2+^ increase following an Fe^2+^ dose-dependent IL-1β production, which results from NLRP3 inflammasome activation (49). Gelfand et al. found that an Fe overload caused retinal degeneration by enhancing the stability of Alu RNAs, thereby promoting retinal pigmented epithelium (RPE) degeneration, thus inducing NLRP3 inflammasome activation (95). Moreover, Liu et al. explored how morphology affects the NLRP3 inflammasome-activating property of iron oxide nanoparticles (IONPs). Research indicates that morphology is a critical determinant of IONP-induced IL-1β release and pyroptosis, and this process is not all mediated by NLRP3 (96).

### 3.6 Manganese

Manganese (Mn) is an essential metal required for proper immune function, regulation of blood sugar and cellular energy, reproduction, digestion, bone growth, blood coagulation and hemostasis, and defense against ROS (27). Mn is used as an additive of biomaterials because of its advantages in stabilizing and promoting osteoblast differentiation and bone metabolism (97). In biological systems, Mn exists in two oxidation states, Mn^2+^ and Mn^3+^, which mediate the redox cycling of Mn and are involved in the biological effects of metals, including the Fenton reaction, transferrin-mediated transport, and interference, as well as interference with other divalent metals (98). In addition, Mn forms various Mn-dependent enzymes, including oxidoreductases, isomerases, transferases, ligases, lyases, and hydrolases (27). Mn^2+^ is also an essential component of some metalloenzymes, such as SOD, glutamate synthetase, pyruvate carboxylase, arginase, hydrolases, phosphatases, transferases, dehydrogenases, kinases, peptidases, and decarboxylases (99). However, excessive levels of Mn can cause cellular toxicity, which leads to oxidative stress, genotoxicity, membrane perturbation, and protein dysfunction by catalyzing the conversion of hydrogen peroxide (H_2_O_2_) into oxygen radical species *via* the Fenton reaction (99). There are several Mn^2+^ importers in the plasma membrane, including SLC39A14, SLC39A8, SLC30A10, and DMT1. In addition, NRAMP1 transports Mn^2+^ from the phagosome to the cytoplasm (99, 100).

Excessive Mn exposure can activate the NLRP3 inflammasome in the microglia, the principal central nervous system (CNS) immune cells, and may result in neurodegenerative disorders. Mn^2+^ activated the NLRP3 inflammasome in the striatum of adult rats and induced the release of ROS, triggering the NLRP3 inflammasome (50). In addition, Mn exposure also resulted in mitochondrial defects that drove the NLRP3 inflammasome signal amplification and propagation and the exosomal release of ASC in microglial cells (51). Another study also showed that Mn^2+^ binding on prion fibrils was critical to inducing the priming signal in NLRP3 inflammasome activation in the microglia (52). Sodium *para*-aminosalicylic acid inhibited Mn-induced NLRP3 inflammasome by inhibiting NF-κB activation and oxidative stress in the microglia (101, 102). On the other hand, the SIRT3 activating enzyme manganese superoxide dismutase (MnSOD) in the mitochondria significantly enhanced the ability to scavenge ROS and suppressed the activation of the NLRP3 inflammasome to protect the heart against oxidative stress (53). Anakinra, the recombinant form of the IL-1 receptor antagonist, dampened the NLRP3 activity by increasing the MnSOD protein longevity (103). Manganese tetrakis porphyrin chloride (MnTBAP), a mitochondrial SOD2 mimic, suppressed NLRP3 inflammasome activation by blocking the albumin-induced mitochondrial dysfunction in renal tubular injury (54). MnTE-2-PyP, another artificial mitochondrial SOD2 mimic, was also proven to suppress the pulmonary hypertension-induced NLRP3 inflammasome activation in macrophages (55). Mn-TAT PTD-Ngb, an artificial metalloprotein containing a TAT protein transduction domain (TAT PTD), which was recombined from apo-neuroglobin (apo-Ngb) with Mn porphyrin, suppressed both NF-κB and ROS-NLRP3 inflammasome activation in the microglia (27, 57).

To date, the role of Mn in NLRP3 inflammasome activation has been examined mainly in relation to the brain neuroscience field. The effect of Mn and the Mn-dependent enzymes on NLRP3 inflammasome activation in the bone healing process needs further exploration, which is beneficial to the application of Mn in bone tissue engineering.

### 3.7 Potassium

Potassium (K) is an essential element in the human body. As a monovalent cation, the potassium ion directly controls other ionic signaling pathways by regulating the membrane potential. In addition, the balance between the intracellular and extracellular fluids is maintained by Na^+^/K^+^ ATPase, which pumps sodium ion (Na^+^) to extra cells while it uptakes K^+^ into the cell in reverse concentration gradients (27, 104).

Activating the NLRP3 inflammasome inevitably leads to potassium efflux, which can also induce NLRP3 inflammasome activation. The molecular mechanisms underlying NLRP3 inflammasome activation through potassium efflux have been extensively investigated. In this review, we only briefly summarized the main mechanisms. For details of the studies, please refer to an elegant review published previously (59). Two potential mechanisms are acknowledged. Potassium efflux may be related to the interaction of NLRP3–NEK7, which is essential to the activation of the NLRP3 inflammasome (58). Another theory is that potassium efflux might promote NLRP3 inflammasome activation by inducing mitochondrial dysfunction and mitochondrial ROS (mtROS) production (105). P2X7 has been reported to be expressed in immune cells such as macrophages, lymphocytes, mast cells, and neutrophils. P2X7 appears to play a critical role in inflammation and autoimmune diseases (82). Extracellular ATP can activate the P2X7 receptor and lead to the activation of the NLRP3 inflammasome. Furthermore, it has also been reported that the non-canonical inflammasome caspase-11 can cleave pannexin-1, followed by ATP release, P2X7 receptor activation, potassium efflux, and NLRP3 inflammasome activation (60). Another non-canonical inflammasome activating the NLRP3 inflammasome mechanism is potassium efflux through the GSDMD-NT-forming pores induced by non-canonical inflammasomes, which further activates the NLRP3 inflammasome (61). The two-pore domain potassium (K2P) is responsible for maintenance of the resting membrane potential in almost all cells. It has been suggested to cooperate with the P2X7 receptor mechanistically (59).

### 3.8 Cobalt

Cobalt (Co) is essential for humans in the form of cobalamin (coenzyme B12), which is tightly bound to a corrin ring and serves as a methyl group carrier with Co oxidation states (106). Co-based alloys are considered one of the most successful materials used for implants as they have satisfactory corrosion, wear, and mechanical properties (107). The homeostasis of Co is maintained by Co^2+^ transporter proteins, including CbiMNQO, NiCoT, HupE/UreJ, CorA, and TBDT. Human serum albumin is considered the primary transporter of Co^2+^ in the blood (108). Although humans are exposed to Co^2+^, the most stable form under ambient conditions, in the course of normal nutrition (109), a high level of Co^2+^ is toxic to cells (104). Co^2+^ has also been reported to induce an immune response by stimulating TLR4 (110).

Exposure to high levels of CoCl_2_ significantly increased the NLRP3 expression, caspase-1 activity, and IL-1β secretion (64). Although a high CoCl_2_ level can induce apoptosis in T lymphocytes, CoCl_2_-treated monocytes did not undergo apoptosis as the effect of p53 was counteracted by the anti-apoptotic activity of the activation of NF-κB and the inflammasome danger signaling pathway leading to the production of pro-inflammatory cytokines (111). However, a study demonstrated that CoCl_2_-induced hypoxia may negatively regulate the NLRP3 inflammasome signaling in brain glial cells (65). Feng et al. found that exposure to Co nanoparticles (Nano-Co) promoted intracellular oxidative stress damage and mtROS, which activated the NLRP3 inflammasome in hepatocytes, suggesting an essential role of the ROS/NLRP3 pathway in Nano-Co-induced hepatotoxicity (66). A study also showed that hemin and cobalt protoporphyrin (CoPP) inhibited NLRP3 inflammasome assembly by reducing the amount of intracellular ASC in cultured macrophages (67).

### 3.9 Chromium

Chromium (Cr), which belongs to the group of trace elements, exists in many different oxidation states in the environment and is essential in numerous functions of the human body. Cr deficiency can cause various physical dysfunctions, while exposure to Cr at higher concentrations is also toxic and may lead to neoplastic diseases. The Co–Cr–Mo alloy is the most widely used combination of Co-based alloys due to its unique combination of strength and ductility (107). Cr(VI) and Cr(III) are the most stable forms of Cr. Cr(VI), the most cancer-related among all Cr oxidation states, enters cells through the sulfate anion transporter system and is reduced to the intermediate oxidation states, e.g., Cr(V) and Cr(IV), in the process of forming stable Cr(III) forms (112).

Adam et al. found the indirect effects of Cr(VI) compounds in pro-inflammation activation. Cr(VI) compounds can induce NLRP3 inflammasome activation and IL-1β production, amplifying the innate immune activation inflammatory response. The authors also confirmed the production of mtROS upstream of the NLRP3 inflammasome assembly by treatment with NAC, suggesting that Cr(VI) induces the production of mtROS and thus activates the NLRP3 inflammasome. In addition, the Cr(III) compounds were also examined. However, the Cr(III) compounds failed to induce these reactions in cells, suggesting that oxidation state-specific differences in mitochondrial reactivity may determine the activation of the inflammasome (62). Jämsen et al. observed that Cr particles alone were insufficient to induce NLRP3 inflammasome activation. Priming human primary macrophages with LPS and exposing the cells to Cr particles were discovered to induce the production of IL-1β, which was significantly reduced by the NLRP3 inflammasome or cathepsin B inhibitor, suggesting that Cr-induced NLRP3 activation is lysosome/cathepsin B-dependent (63). In addition, Cr^3+^ can activate both priming signaling and activation signaling of the NLRP3 inflammasome by inducing ROS accumulation (28).

### 3.10 Nickel

Nickel (Ni) is an abundant element in the earth’s core and is a commonly used implant material as it grants necessary strength and durability to the implant. However, it is also associated with metal hypersensitivity reactions and can be found in trace amounts in “commercially pure” Ti materials used in surgical implants (113, 114). The uptake of Ni has a toxic effect on cell metabolism and physiology in humans. The toxicity of Ni is dependent on the solubility of the Ni compounds. Insoluble Ni compounds are phagocytosed by cells, while Ni ions are delivered into cells and induce the production of free radicals (68). Ni^2+^ triggers an inflammatory response by activating human TLR4 (115).

Xin et al. found that Ni-refining fume particles can induce the decrease of the mitochondrial membrane potential (MMP) and the increase of the opening rate of the mitochondrial permeability transition pore (MPTP) in human lung epithelial BEAS-2B cells, and activation of the NLRP3 inflammasome induced by Ni-refining fume particles can be significantly suppressed by NAC, an effective ROS remover, suggesting that Ni-refining fume particles activate the NLRP3 inflammasome by causing mitochondrial dysfunction and ROS production (68). Another study also showed that Ni-refining fumes promoted the expression of the NLRP3 inflammasome by inducing the Warburg effect in BEAS-2B cells (69). In addition, it has also been confirmed that activation of the NLRP3 inflammasome by Ni ions is independent of the phagolysosome–cathepsin B pathway (70). However, in lung pathology, Ni-contaminated particles activated the NLRP3 inflammasome by disrupting macrophage phagolysosomes, which resulted in prolonged inflammation (71). In a study of Ni ions, NiCl_2_ induced the accumulation of ROS and mitochondrial DNA, resulting in the activation of the NLRP3 inflammasome. It was also found that NiCl_2_-induced apoptosis is dependent on ROS generation, suggesting that NiCl_2_ can induce both apoptosis and pyroptosis (72).

### 3.11 Metal element-bearing ceramic materials

Ceramic materials are widely used in orthopedic implantations because of their low osteolytic potential and friction coefficients and high biocompatibility. The particles of ceramic materials can be taken up by immune cells and can induce an immune response, including inflammasome activation (31, 116). Titanium dioxide (TiO_2_), zirconium oxide (ZrO_2_), and aluminum oxide (Al_2_O_3_) are widely applied in bioceramic implantation due to their satisfactory properties of wear, tear, hardness, biocompatibility, and corrosion resistance (107). Cytotoxicity significantly increases when macrophages are exposed to high concentrations of ZrO_2_ particles (≥10^7^ particles/ml). However, compared to TiO_2_, ZrO_2_ particles produce less inflammatory cytokines, suggesting that they are less toxic than TiO_2_ (117). Jamieson et al. found that Al_2_O_3_ and ZrO_2_ treatment can enhance the gene expression of IL-1β, which is TLR4-dependent. Priming cells with LPS following Al_2_O_3_ or ZrO_2_ treatment can induce cell death. In addition, LPS and ZrO_2_ treatment can also induce IL-1β protein secretion, while treatment with LPS and Al_2_O_3_ was insufficient to induce it. These results suggest that ZrO_2_, but not Al_2_O_3_, may activate the inflammasome (73). In another *in vivo* study, intraperitoneal injection of a water-soluble supernatant with Al_2_O_3_ in mice revealed that the mRNA expression of NLRP3 decreased while that of caspase-1 did not change (74).

## 4 Conclusion and perspective

Metals are widely used in the fabrication of implants due to their advantage of having good mechanical properties and ductility compared to other biomaterials. However, metal corrosion inducing ion release and metal debris production is inevitable. At the same time, the amount of metal released is highest after the operation, which is also the acute inflammatory phase initiated by injury to the tissue and is one of the factors that define the outcome of the implant (118). A growing number of studies has provided new insights into how these metals affect the early inflammatory response of bone regeneration after metal implantation. However, whether the specific ion activates inflammasomes synergistically or singly is still unclear. The detailed mechanisms of metal ions activating inflammasomes are still under investigation. Additional studies are also needed to further understand the roles of metal implantation debris and the metal ions released from implantation in mediating the process of inflammasome activation. Immunomodulatory alloy biomaterials based on the NLRP3 inflammasome activation mechanism could be developed, but the immune response of tissues to these biomaterials needs to be further confirmed.

## Author contributions

WH, ZZ, and YQ wrote the manuscript and created the figures and the table. QZ and QW reviewed and edited the manuscript and provided guidance. YG and YF provided important perspective of the manuscript. All authors contributed to the article and approved the submitted version.

## Glossary

**Table:**

AIM2	melanoma 2
ASC	apoptosis-associated speck-like protein containing a CARD
bBox	zinc finger domain
BMDMs	bone marrow-derived macrophages
CARD	caspase recruitment domain
CTR1	copper transporter 1
DMT1	divalent metal ion transporter 1
DAMPs	damage-associated molecular patterns
FL-GSDMD	full-length gasdermin D
GSDMD-NT	N-terminal GSDMD
HPMCs	human peritoneal mesothelial cells
HEK	human embryonic kidney
LPS	lipopolysaccharide
LRR	leucine-rich repeat
MGL	magnesium isoglycyrrhizinate
MMP	mitochondrial membrane potential
MnTBAP	Mn tetrakis porphyrin chloride
MnSOD	manganese superoxide dismutase
MPTP	mitochondrial permeability transition pore
mPTCs	mouse proximal tubular cells
mtROS	mitochondrial reactive oxygen species
NOD	nucleotide-binding oligomerization domain
NLR	NOD-like receptor
NLRP1	NLR family pyrin domain containing 1
NEK7	NIMA-related kinase 7
NLRC4	NAIP-NLR family caspase-associated recruitment domain-containing protein
PAMPs	pathogen-associated molecular patterns
PBMCs	peripheral blood mononuclear cells
PRRs	pattern recognition receptors
PYD	pyrin domain
rMSCs	rat mesenchymal stem cells
ROS	reactive oxygen species
SOD	superoxide dismutase
TNF-a	tumor necrosis factor alpha
TPEN	*N,N,N′,N′*-tetrakis (2- pyridylmethyl)ethylenediamine
TTM	tetrathiomolybdate
TF	transferrin
ZnONPs	zinc oxide nanoparticles
